# Longitudinal Changes of Functional Capacities Among Adolescent Female Basketball Players

**DOI:** 10.3389/fphys.2019.00339

**Published:** 2019-04-04

**Authors:** Humberto M. Carvalho, Thiago J. Leonardi, André L. A. Soares, Roberto R. Paes, Carl Foster, Carlos E. Gonçalves

**Affiliations:** ^1^Department of Physical Education, School of Sports, Federal University of Santa Catarina, Florianópolis, Brazil; ^2^Physical Education School, Federal University of Rio Grande do Sul, Porto Alegre, Brazil; ^3^Faculty Physical Education, University of Campinas, Campinas, Brazil; ^4^Department of Exercise and Sport Science, University of Wisconsin-LaCrosse, LaCrosse, WI, United States; ^5^Faculty of Sport Sciences and Physical Education, University of Coimbra, Coimbra, Portugal

**Keywords:** youth sports, menarche, athletes, Bayesian multilevel modeling, adolescence

## Abstract

**Background:** The interpretation of young athletes' performance during pubertal years is important to support coaches' decisions, as performance may be erroneously interpreted due to the misalignment between chronological age (CA), biological age (BA) and sport age (SA).

**Aim:** Using a Bayesian multilevel approach, the variation in longitudinal changes in performance was examined considering the influence of CA, BA (age at menarche), SA, body size, and exposure to training among female basketball players.

**Method:** The study had a mixed-longitudinal design. Thirty eight female basketball players (aged 13.38 ± 1.25 years at baseline) were measured three times per season. CA, BA and SA were obtained. Anthropometric and functional measures: countermovement jump, Line drill (LD), Yo-Yo (Yo-Yo IR1). Based on the sum of the z-scores, an index of overall performance was estimated. The effects of training on longitudinal changes in performance were modeled.

**Results:** A decrease in the rate of improvements was apparent at about 14 years of age. When aligned for BA, the slowing of the rate of improvements is apparent about 2 years after menarche for LD. For countermovement jump longitudinal changes, when performance was aligned for BA improvements became linear. For Yo-Yo IR1 and performance index, both indicators showed a linear trend of improvement when aligned for CA and BA, separately. Older players showed higher rates of improvement for Yo-Yo IR1 and performance index from pre-season to end-season. When considering performance changes aligned for BA it was apparent an improvement of performance as players became biologically mature.

**Conclusions and Implications:** The alignment of CA with BA and SA provides important information for coaches. Human growth follows a genetically determined pattern, despite variation in both tempo and timing. When the effects of maturation reach their end, all the girls went through the same process. Hence, there is no need to artificially manipulate youth competitions in order to accelerate gains that sooner or later reach their peak and tend to flat their improvement curve.

## Introduction

The interpretation of young athletes' performance development during pubertal years is of importance to support coaches' short- and long-term decisions. Particularly in the context of talent development where, despite ethical issues, early identification and selection is the *modus operandi* of high performance sport, talent selection or de-selection decisions have different levels of risks and consequences for young players (Baker et al., 2018).

Most notably, performance assessment may be confounded due to the misalignment between chronological age, biological age and sport age (accumulated training and competitive experience in sport). Therefore, the mechanisms that predict future successful players and those dropping out from organized sports are multifactorial and highly complex, especially in sports like basketball, where structured training systems start before or during puberty (Deprez et al., 2015; Soares et al., 2016; Gonçalves et al., 2018).

Basketball is a team sport that requires movement patterns that involve short, intense and repeated episodes of activity requiring frequent rapid changes in direction (Mcinnes et al., 1995; Ben Abdelkrim et al., 2007, 2010; Staunton et al., 2018; Stojanovic et al., 2018). Although basketball movement patterns mainly involve intermittent activities which are aerobic in nature, maximal intensity short-term activities (e.g., sprinting, jumping, cutting) are decisive for the performance in the game (Ben Abdelkrim et al., 2007; Stojanovic et al., 2018). Hence, when interpreting young basketball player's performance, coaches and/or researchers should consider both maximal short-term output and basketball related intermittent endurance. On the other hand, body size, particularly stature, is relevant for basketball performance (Drinkwater et al., 2008), and is highly valued by coaches when attempting to select and/or predict future outcomes (Pearson et al., 2006). However, young players vary substantially in growth and maturity status, as well as complex environmental factors, often complicates interpretation of performance in young athletes (Abbott et al., 2005; Pearson et al., 2006).

There is an emphasis in youth sports, including youth basketball, on talent identification, development and selection (Gonçalves et al., 2012). Achievement of athletic expertise at high level when adults is limited to a very narrow group of players.

In youth basketball, coaches' decisions regarding the career path of adolescent players are influenced by players size and functional performance level (Drinkwater et al., 2008; Carvalho et al., 2018). Although differences in adolescent players' physique and performance are transient, are likely exacerbated by the interactions between pubertal growth rate, chronological age and accumulated sport-specific experience (Carvalho et al., 2018; Gonçalves et al., 2018; Leonardi et al., 2018). Thus, the appropriate interpretation of the young basketball players' performance is crucial for both coach and athlete (Leonardi et al., 2018).

Available data considering functional capacities in young basketball players is mainly based on male players (Montgomery et al., 2008; Carvalho et al., 2011a,b,c; Sisic et al., 2016; Torres-Unda et al., 2016). Although there is an increasing number of female young athletes involved in intensive training programs and high level competitions, available knowledge concerning the functional capacities of young female basketball players remains scarce (Mcmanus and Armstrong, 2011). Interpretations of young female athletes' functional capacity may be complicated by sexual dimorphism (Mcmanus and Armstrong, 2011), given the large variation between-girls in the timing and tempo of biological maturation, as well as primary sex differences (Sherar et al., 2004). Hence, interpretations based on male athlete samples may be inadequate and examining functional capacity in young female athletes engaged in sport-specific training merits attention.

Understanding changes and development of performance during pubertal development is an increasingly studied topic (Nevill et al., 1998; Thomis et al., 2000; Beunen et al., 2002; De Ste Croix et al., 2002; Martin et al., 2004; Drinkwater et al., 2005; Bidaurrazaga-Letona et al., 2014; Carvalho et al., 2017). Amongst young athletes exposed to organized training and competition programs, researchers usually need a long time planning, extensive resources for data collection in the field, rather than the laboratory. Attrition from injuries or loss of interest complicates data analysis. Furthermore, the need to consider chronological age, biological age and “the age in the sport” (i.e., the amount of accumulated training and competition experience in the sport) represent a level complexity that may be difficult to appropriately fit and interpret using traditional statistical models (i.e., based on repeated measures analysis of variance) (Gueorguieva and Krystal, 2004; Kristensen and Hansen, 2004). Multilevel modeling provides a flexible and powerful approach to fit complex hierarchical structured data, such as repeated measures (Gelman and Hill, 2007; Goldstein, 2011). Furthermore, Bayesian methods are especially attractive in this context, as they perform well with small sample sizes (Van De Schoot et al., 2015), perform well with complex models such as multilevel modeling (McElreath, 2015), and allow incorporation of available prior information about the parameters in evaluating the data consequently improving out-of-sample predictions (Heino et al., 2018).

Considering a Bayesian multilevel approach, we examined the influence of chronological age, biological age (age at menarche), age in the sport, body size and composition, and exposure to training and competitive basketball season on the longitudinal changes in functional performance during the pubertal years among female basketball players.

## Methods

### Study Design and Participants

This study was based on a mixed-longitudinal design. A total of 38 adolescent female basketball players aged, on average, 13.38 (1.25) years at baseline, were measured three times per season between August 2015 and December 2017. Within the season, measurements were performed pre- (March), mid- (August) and end-season (December). The period of observation comprised two full competitive seasons (from March 2016 to December 2017), and a half season (August 2015 to December 2015). A total of 177 observations were considered for analysis, during the observation period as follows: August 2015, *n* = 10; December 2015, *n* = 9; March 2016, *n* = 31; August 2016, *n* = 37; December 2016, *n* = 35; March 2017, *n* = 24; August 2017, *n* = 17; December 2017, *n* = 14). The players considered in the present study had at least three measurements across the period of observation. The distribution of measurements per players was as follows: three measurements, *n* = 6; four measurements, *n* = 10; five measurements, *n* = 13; six measurements, *n* = 9). All The players were engaged in formal training and competition within under 13 (*n* = 23) and under 15 (*n* = 15) teams from two clubs from the Campinas metropolitan region of Brazil, and competed at regional level competition supervised by the *Associação Regional de Basquetebol* (ARB). During the study, all players trained regularly (~300–360 min/wk) over a 9-month season (March to November). The typical week was composed by three training sessions, with a duration of 120 min per session. In general, sessions were composed by a warm-up section (~30 min), an individual technical development section (~30 min), tactical development section (~30 min, mostly small sided games), and game session (~30 min). No player was suffering from injury at the time of testing or during 6 months before testing.

The study was approved by the Research Ethics Committee of the University of Campinas. Participants were informed about the nature of the study, that participation was voluntary and that they could withdraw from the study at any time. Players and their parents/legal guardians provided written informed consent.

### Measures

Chronological age was calculated to the nearest 0.1 year by subtracting birth date from date of testing. Years of training in formal basketball were attained by interview. Age at menarche was obtained from an individual interview by the coaches of the players (female coaches in all cases). To align age at menarche with chronological age (i.e., distance to menarche) we subtracted chronological age by age at menarche. Negative values indicate time before age at menarche and positive values indicate time after age at menarche.

Anthropometric measurements were performed by a single experienced observer. Stature was measured with a portable stadiometer (Seca model 206, Hanover, MD, USA) to the nearest 0.1 cm. Body mass was measured with a calibrated portable balance (Seca model 770, Hanover, MD, USA) to the nearest 0.1 kg. The triceps, subscapular, suprailiac and medial calf skinfolds were measured and summed as a measure of relative body fat distribution. Skinfold sites were measured with a Lange skinfold caliper (Cambridge Scientific Industries, Inc., Cambridge, MD). Reliability estimates for the observer are published elsewhere (Carvalho et al., 2011a,b).

Three measures of functional capacity for basketball were considered: vertical jump with countermovement (Bosco et al., 1983), a short-term maximal running protocol, the Line drill (LD) test (Semenick, 1990; Carvalho et al., 2011a) and an intermittent endurance test, the Yo-Yo Intermittent Recovery level 1 test (Yo-Yo IR1) (Bangsbo, 1994). Based on the sum of the z-scores, we estimated an index of overall performance, i.e., functional performance index (lower-limb explosive strength, agility and anaerobic power, and intermittent endurance). Note that z-scores were reversed for the LD performance; as lower times indicate better performance.

Tests were performed in two sessions separated by at least 48 h, where the first session included the vertical jump and LD test, and the second session the Yo-Yo IR1. Before testing a standardized warm-up was taken by all athletes.

The countermovement jump test was tested on a jump mat (Multisprint System, Hidrofit, Brazil**)**. Participants started from an upright standing position. Players were instructed to begin the jump with a downward movement, which was immediately followed by a concentric upward movement, resulting in a maximal vertical jump. During jumping, hands were held on the hips during all phases of the jumping. Three trials were allowed and the best retained for analysis. The coefficient of variation, based on replicate measures separated by 1 week in 18 players, was 6.9% (95% CI 5.1–10.5).

In the LD protocol (Semenick, 1990; Carvalho et al., 2011a), players ran 140 m as fast as possible in the form of four consecutive shuttle sprints of 5.8, 14.0, 22.2, and 28.0 m within a regulation basketball court. Players began the test one meter behind the baseline of the basketball court, where a pair of photoelectric cells (Multisprint System, Hidrofit, Brazil**)** was aligned with the baseline. Verbal encouragement for an all-out effort was given throughout the test. Time was recorded in seconds. Reliability estimates were reported previously (Carvalho et al., 2011a).

The Yo-Yo IR1 was performed by all players (Bangsbo, 1994). The protocol is based on repeated 2 x 20-m runs back and forth between the starting, turning, and finishing line at a progressively increased speed controlled by audio bleeps from a tape recorder (Bangsbo, 1994). The athletes have a 10-s active rest period between each bout, jogging in a distance of 2 × 5-m. Players ran until they were no longer able to maintain the required speed; the test was completed when athletes failed twice to reach the finishing line in time. Covered distance was measured in meters. Based on replicate measures on a subsample of 11 players measured twice within 1 week, the coefficient of variation was 6.0% (95% CI 4.5–9.5%), which is within the range of reproducibility reported for the Yo-Yo IR1 (Bangsbo et al., 2008).

### Statistical Analysis

#### Modeling Functional Performance Aligned by Chronological Age or Age at Menarche

The first modeling step was to use a basic two-level polynomial growth model curve (Goldstein, 1986) to model functional performance indicators against chronological age and age at menarche, i.e., distance to menarche in years, separately. The model describes each player's successive measurements over time defining the player's change at each measurement point and its variation (level-1), differences in trajectories between players and its variation (level-2). To capture the possibility of non-linear longitudinal changes during pubertal years we considered time (chronological age or distance to menarche) coefficients up to the quadratic terms, at least. When modeling functional capacity indicators against chronological age we centered each player's value at the sample grand mean (13.94 years). This allows for the model to provide predicted values with meaningful information within the range of observations, in particular the intercept term. We allowed for between-participants variation at group-level (level-2) across the intervals of observations.

Since both time indicators were centered, we used weakly informative prior distributions for population-level, normal priors (0.50), and for group-level effects, half-cauchy priors (0.2). This conveniently allows for easier achievement of model convergence, as well as ensuring that results reflect the knowledge available from the current data.

#### Modeling the Influence of Body Size and Training Experience on Functional Performance Longitudinal Changes

In this step of the analysis we explored whether body size (stature, body mass and adiposity represented by the sum of four skinfolds) and years formal training experience influenced longitudinal changes in functional performance. For computational convenience and for interpretation when variables have different scales (McElreath, 2015) we used z-score transformation on both dependent variables (functional performance indicators) and independent variables (i.e., the candidate explanatory variables training experience, stature, body mass and adiposity). We added the explanatory variables to each of the basic two-level polynomial growth model modeling functional performance indicators against chronological age and distance to menarche. In the models for this step we also used weakly informative prior distributions for population-level, normal priors (0.10), and for group-level effects, half-cauchy priors (0.2).

#### Modeling the Effects of Exposure to 9-Months Competitive Season on Longitudinal Changes in Functional Performance

Based on the results of the previous analytical step, we explored the effects of training exposure on longitudinal changes in functional performance with indicators aligned for years of formal training in basketball, controlling for chronological age and distance to menarche. Since measurements were made and pre-, mid- and end-season across two and a half years, we first examined the pattern of change within the 9-month competitive season. A linear trend of change was observed, thus we included a dummy variable (pre-season coded as 0; mid-season coded as 1; end-season coded as 2) to identify each moment of observation in the models. We then included the dummy variable (i.e., season) in each of the initial models predicting functional performance indicators against chronological age and distance to menarche in years. We also considered an interaction term of the time variable with the dummy variable (e.g., distance to menarche interaction with season). The inclusion of the dummy variable for season and the interaction terms allow us to examine whether there are differences in players' functional performance indicators rates of change within the 9-month season, as well as changes within the 9-month season change with chronological age, distance to menarche or years of training experience (interaction term between time of measurement and season measurement).

Similar to the precedent models, we used weakly informative prior distributions for population-level, normal priors (0.10), and for group-level effects, half-cauchy priors (0.2), allowing model convergence, as well as ensuring that results reflect the knowledge available on the current data.

#### Model Checking and Computation

We used posterior predictive checks to confirm that we did not omit relevant interactions (Gelman et al., 2013; Vehtari et al., 2016). We used the widely applicable information criteria (WAIC) to compare models and to ensure we had not overfit our data (Gelman et al., 2013; McElreath, 2015; Vehtari et al., 2016).

For each model we run two chains for 2,000 iterations with a warm-up length of 1,000 iterations. The models were implemented with Bayesian methods via Markov Chain Monte Carlo (MCMC) simulation and using Hamiltonian Monte Carlo and its extension, the No-U-Turn Sampler using Stan (Stan Development Team, 2018), via “brms” package (Bürkner, 2017), available as a package in the R statistical language (R Core Team, 2015).

## Results

**Figure 1:**
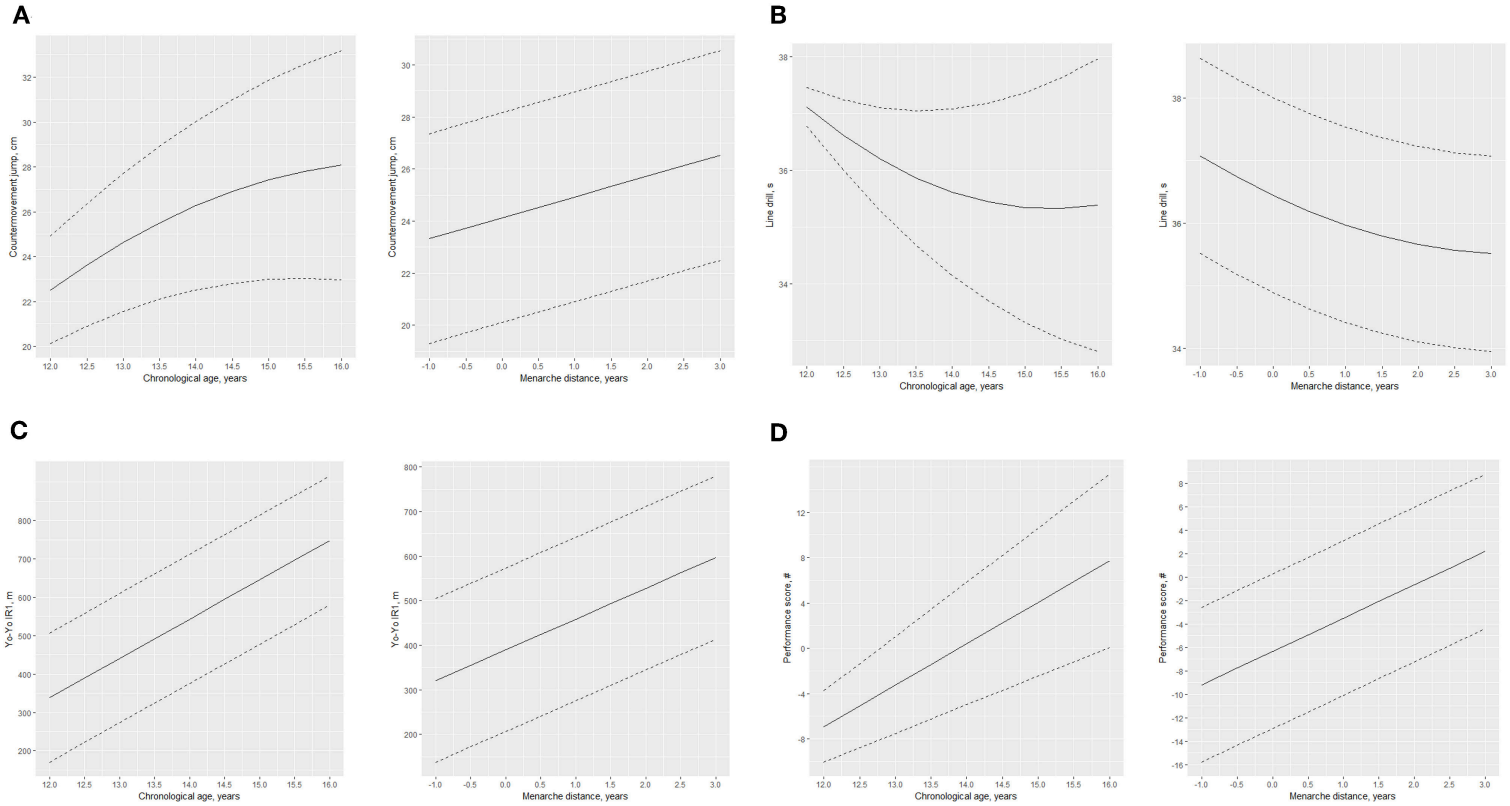
Countermovement jump **(A)**, Line drill test **(B)**, Yo-Yo IR1 **(C)**, and performance index **(D)** of young female basketball players by chronological age and by menarcheal status.

The average age at menarche for the present sample of adolescent female basketball players was 11.82 (1.25) years. Five players attained menarche during the study. The posterior predictions and 90% credible intervals for indicators of functional performance aligned by chronological age and distance to age at menarche of young Brazilian female basketball players are summarized in Table 1 and Figure 1. Corresponding Bayesian multilevel models from where posterior samples were derived are summarized in Supplementary Tables 1, 2. A non-linear trend was observed for both countermovement jump and Line drill performances when aligning performance by chronological age. A decrease in the rate of improvements in both jump and Line drill changes was apparent at about 14 years of age. When aligned for distance to menarche, the slowing of the rate of improvement was apparent about 2 years after menarche for Line drill performance. As for jump performance longitudinal changes, when performance was aligned for age at menarche improvements became linear. For Yo-Yo IR1 and functional performance index, both indicators showed a linear trend of improvement in performance when aligned for chronological age and distance to menarche. For all functional performance indicators except Yo-Yo IR1, variation between players in longitudinal changes was substantial when aligned for chronological age (see Supplementary Table 1). However, between-player variation was not apparent when functional performance was aligned for age at menarche (see Supplementary Table 2).

**Table 1:** Posterior predictions and 90% credible intervals for longitudinal changes of functional performance aligned both by chronological age and age at menarche.

	**Countermovement jump, cm**	**Line drill test, s**	**Yo-Yo IR1, m**	**Performance index, #**
12 years	22.52 (21.56 to 23.50)	37.11 (36.72 to 37.51)	338.3 (330.6 to 347.20)	−6.90 (−7.16 to −6.65)
13 years	24.63 (23.81 to 25.46)	36.20 (35.91 to 36.51)	440.7 (418.9 to 463.3)	−3.25 (−3.96 to 2.51)
14 years	26.26 (25.08 to 27.41)	35.61 (35.13 to 36.31)	543.1 (490.6 to 596.0)	0.40 (−1.27 to 2.13)
15 years	27.41 (25.41 to 29.38)	35.34 (34.36 to 36.34)	645.5 (562.3 to 728.7)	4.05 (1.42 to 6.79)
16 years	28.09 (24.77 to 31.34)	35.39 (33.61 to 37.19)	747.9 (634.0 to 861.4)	7.70 (4.11 to 11.44)
1 year before age at menarche	23.33 (22.11 to 24.45)	37.07 (36.74 to 37.41)	320.9 (264.8 to 370.0)	−9.18 (−11.14 to −6.82)
Age at menarche	24.13 (22.35 to 25.81)	36.44 (35.73 to 37.11)	389.8 (306.5 to 466.5)	−6.34 (−9.28 to −3.28)
1 year after age at menarche	24.93 (22.59 to 27.17)	35.97 (34.72 to 37.17)	458.7 (348.2 to 563.0)	−3.50 (−7.42 to 0.26)
2 years after age at menarche	25.73 (22.83 to 28.53)	35.66 (33.71 to 37.59)	527.6 (389.9 to 659.5)	−0.66 (−5.56 to 3.8)
3 years after age at menarche	26.53 (23.07 to 29.89)	35.51 (32.70) to 38.37)	596.5 (431.6 to 756.0)	2.18 (−3.7 to 7.34)

The relative contributions of formal experience of training, stature, body mass and adiposity on the longitudinal changes in functional performance indicators aligned for chronological age are summarized in Table 2, and aligned for age at menarche in Table 3. Adiposity had a negative influence on players' functional performance. Between-player differences in body mass did not influence longitudinal changes in functional performance, whether performance was modeled against chronological age or age at menarche.

**Table 2:** Relative contributions of years of formal experience of basketball training, body size and adiposity on longitudinal changes in functional performance aligned by chronological age.

	**Countermovement jump**	**Line drill test**	**Yo-Yo IR1**	**Performance index**
**POPULATION-LEVEL EFFECTS (90% CREDIBLE INTERVAL)**				
Intercept	−3.38 (−12.37 to 5.56)	3.10 (−3.92 to 10.40)	−5.25 (−41.22 to 30.22)	−14.85 (−54.58 to −26.03)
Chronological age	0.34 (0.00 to 0.70)	−0.04 (−0.29 to 0.22)	2.74 (1.47 to 4.09)	2.77 (1.27 to 4.40)
Years of formal training experience	0.39 (0.04 to 0.72)	−0.36 (−0.60 to −0.12)	0.68 (−0.61 to 2.01)	1.65 (0.13 to 3.14)
Years of formal training experience^2^	−0.18 (−0.28 to −0.07)	0.10 (0.01 to 0.20)	–	–
Stature	−0.09 (−0.13 to 0.31)	−0.08 (−0.26 to 0.09)	0.14 (−0.74 to 1.03)	0.38 (−0.63 to 1.37)
Body mass	−0.18 (−0.52 to 0.16)	0.12 (−0.13 to 0.38)	0.53 (−0.94 to 2.01)	0.53 (−1.08 to 2.14)
Sum of four skinfolds	−0.24 (−0.54 to 0.08)	0.18 (−0.03 to 0.39)	−1.91 (−3.31 to −0.54)	−2.60 (−4.11 to −1.05)
**GROUP-LEVEL EFFECTS (90% CREDIBLE INTERVAL)**				
**Level 1 standard deviation (within player)**				
Within-individuals	0.71 (0.63 to 0.80)	0.72 (0.63 to 0.81)	3.65 (3.26 to 4.09)	3.80 (3.35 to 4.25)
**Level 2 standard deviation (between players)**				
Intercept	1.06 (0.80 to 1.38)	0.48 (0.11 to 0.78)	3.61 (2.56 to 4.76)	4.07 (2.79 to 5.55)
Chronological age	0.25 (0.02 to 0.57)	0.31 (0.02 to 0.70)	1.10 (0.13 to 2.27)	1.05 (0.07 to 2.41)
Years of formal training experience	0.24 (0.02 to 0.56)	0.32 (0.03 to 0.69)	1.11 (0.12 to 2.31)	1.47 (0.23 to 2.74)

*variables were standardized*.

**Table 3:** Relative contributions of years of formal experience of basketball training, body size and adiposity on longitudinal changes in functional performance aligned by age at menarche.

	**Countermovement jump**	**Line drill test**	**Yo-Yo IR1**	**Performance index**
**POPULATION-LEVEL EFFECTS (90% CREDIBLE INTERVAL)**				
Intercept	−5.14 (−13.72 to 3.39)	2.22 (−5.32 to 9.07)	−15.15 (−48.43 to 19.29)	−21.37 (−61.89 to 18.70)
Distance to menarche	−0.10 (−0.44 to 0.27)	0.05 (−0.19 to 0.29)	2.11 (0.76 to 3.52)	2.04 (0.57 to 3.62)
Years of formal training experience	0.69 (0.46 to 0.94)	−0.41 (−0.63 to −0.18)	1.93 (0.81 to 3.10)	2.73 (0.1.52 to 4.01)
Years of formal training experience^2^	−0.19 (−0.29 to −0.08)	0.10 (0.01 to 0.21)	-	-
Stature	0.14 (−0.07 to 0.35)	−0.06 (−0.23 to 0.12)	0.32 (−0.51 to 1.14)	0.48 (−0.51 to 1.48)
Body mass	−0.09 (−0.44 to 0.25)	0.07 (−0.17 to 0.32)	0.43 (−1.03 to 1.94)	0.48 (−1.13 to 2.06)
Sum of four skinfolds	−0.32 (−0.62 to −0.01)	0.19 (−0.01 to 0.40)	−1.99 (−3.47 to −0.61)	−2.58 (−4.16 to −1.02)
**GROUP-LEVEL EFFECTS (90% CREDIBLE INTERVAL)**				
**Level 1 standard deviation (within player)**				
Within-individuals	0.73 (0.65 to 0.82)	0.72 (0.64 to 0.81)	3.67 (3.30 to 4.08)	3.87 (3.42 to 4.37)
**Level 2 standard deviation (between players)**				
Intercept	0.85 (0.36 to 1.33)	0.44 (0.07 to 0.84)	0.97 (0.06 to 2.55)	3.52 (1.19 to 5.57)
Distance to menarche	0.32 (0.04 to 0.69)	0.16 (0.01 to 0.39)	0.76 (0.08 to 1.91)	1.25 (0.09 to 2.96)
Years of formal training experience	0.28 (0.02 to 0.59)	0.39 (0.05 to 0.77)	1.21 (0.73 to 1.75)	1.52 (0.27 to 2.81)

*variables were standardized*.

The results for the model exploring the effects of exposure to 9-months competitive season on longitudinal changes in functional performance are summarized in Table 4, when aligning for chronological age, and Table 5, when aligning for age at menarche.

**Table 4:** Posterior estimates for longitudinal changes in functional performance aligned by chronological age and partitioning the influence of exposure to 10-month competitive seasons.

	**Countermovement jump**	**Line drill test**	**Yo-Yo IR1**	**Performance index**
**POPULATION-LEVEL EFFECTS (90% CREDIBLE INTERVAL)**				
Intercept	−0.01 (−0.35 to 0.34)	0.19 (−0.04 to 0.42)	−1.78 (−2.70 to −0.84)	−2.12 (−3.25 to −0.95)
Chronological age	0.11 (−0.19 to 0.41)	0.14 (−0.08 to 0.36)	1.65 (1.02 to 2.29)	2.14 (1.39 to 2.92)
Years of formal training experience	0.46 (0.16 to 0.77)	−0.42 (−0.70 to −0.16)	-	1.11 (1.42 to 2.86)
Years of formal training experience^2^	−0.17 (−0.26 to −0.08)	0.11 (0.01 to 0.22)	-	-
Season	0.34 (0.22 to 0.45)	−0.36 (−0.47 to −0.24)	1.70 (0.94 to 2.48)	2.14 (1.42 to 2.86)
Season × chronological age interaction	-	-	0.62 (0.14 to 1.10)	0.63 (0.16 to 1.12)
Sum of 4 skinfolds	−0.29 (−0.49 to −0.09)	0.20 (0.04 to 0.36)	−0.97 (−1.65 to −0.28)	−1.62 (−2.45 to −0.75)
**GROUP-LEVEL EFFECTS (90% CREDIBLE INTERVAL)**				
**Level 1 standard deviation (within player)**				
Within-individuals	0.65 (0.58 to 0.74)	0.64 (0.56 to 0.71)	2.93 (2.60 to 3.30)	2.96 (2.60 to 3.37)
**Level 2 standard deviation (between players)**				
Intercept	1.02 (0.78 to 1.31)	0.35 (0.05 to 0.67)	2.37 (1.58 to 3.29)	3.18 (2.18 to 4.35)
Chronological age	0.21 (0.02 to 0.51)	0.43 (0.12 to 0.73)	-	1.10 (0.16 to 2.18)
Years of formal training experience	0.25 (0.02 to 0.61)	0.60 (0.26 to 0.91)	-	-
Season	-	-	2.02 (1.32 to 2.78)	1.69 (0.90 to 2.56)

*variables were standardized*.

**Table 5:** Posterior estimates for longitudinal changes in functional performance aligned by menarche age and partitioning the influence of exposure to 10-month competitive seasons.

	**Countermovement jump**	**Line drill test**	**Yo-Yo IR1**	**Performance index**
**POPULATION-LEVEL EFFECTS (90% CREDIBLE INTERVAL)**				
Intercept	−0.44 (−0.06 to 0.06)	0.03 (−0.39 to 0.32)	−3.82 (−5.37 to −2.32)	−3.57 (−5.57 to −1.70)
Distance to menarche	−0.33 (−0.66 to −0.01)	0.16 (−0.05 to 0.39)	1.51 (0.50 to 2.52)	0.59 (−0.19 to 1.39)
Years of formal training experience	0.69 (0.47 to 0.92)	−0.40 (−0.65 to −0.15)	0.91 (0.19 to 1.63)	1.84 (0.87 to 2.80)
Years of formal training experience^2^	−0.19 (0.27 to −0.10)	0.11 (0.1 to 0.22)	-	-
Season	0.23 (0.06 to 0.41)	−0.36 (−0.47 to −0.15)	-	-
Season × distance to menarche interaction	0.06 (−0.00 to 0.13)	-	0.78 (0.10 to 1.48)	0.85 (0.61 to 1.10)
Sum of four skinfolds	−0.26 (−0.46 to −0.06)	0.20 (0.06 to 0.34)	−1.10 (−1.84 to −0.36)	−1.49 (−2.33 to −0.63)
**GROUP-LEVEL EFFECTS (90% CREDIBLE INTERVAL)**				
**Level 1 standard deviation (within player)**				
Within-individuals	0.66 (0.58 to 0.74)	0.63 (0.56 to 0.72)	2.95 (2.60 to 3.34)	2.94 (2.59 to 3.35)
**Level 2 standard deviation (between players)**				
Intercept	0.86 (1.08 to 2.27)	0.33 (0.05 to 0.64)	2.46 (1.63 to 3.41)	2.83 (1.19 to 4.43)
Distance to menarche	0.22 (0.03 to 0.43)	-	-	1.21 (0.12 to 2.63)
Years of formal training experience	-	0.60 (0.32 to 0.90)	-	-
Season	-	0.11 (0.01 to 0.25)	2.11 (1.36 to 2.96)	1.89 (1.18 to 2.71)

*variables were standardized*.

Considering performance changes aligned for chronological age and accounting for between players' differences in training experience and adiposity, it was observed a trend for substantial improvements of all performance indicators across the 9-months season (i.e., between pre-, mid- and end-season). Also, it was apparent that older players showed higher rates of improvement for Yo-Yo IR1 and functional performance index from pre-season to end-season. Also, when considering performance changes aligned for age at menarche (Table 5), there was an apparent substantial improvement of performance across the season. However, for countermovement jump, Yo-Yo IR1 and functional performance index from pre-season to end season improvements were substantially higher as players became biologically mature (greater distance to menarche).

## Discussion

The present study modeled longitudinal changes in functional performance considering the influence of chronological age, biological age (age at menarche), age in the sport, body size and body composition, and exposure to training and competitive basketball season over the pubertal years in female basketball players. There is a body of literature that addresses the growth curves and functional performance development of pubertal girls (Nevill et al., 1998; Yagüe and De La Fuente, 1998; Armstrong et al., 2000; Thomis et al., 2000; De Ste Croix et al., 2002; Geithner et al., 2004), but mostly in non-athletic populations. A challenging question of interest with young athletes is to understand the complex interactions of growth and development with the exposure to athletic and sport-specific performance. However, to our knowledge, this is the first study that aims to align performance development with chronological age, biological age and sport experience, and to shed light on their relative effect on performance.

Chronological age and biological age are genotype variables and age in the sport is a phenotype one, meaning that the knowledge of the contribution and the interaction of each of the variables to performance in the developmental years is an important issue for researchers and practitioners. The findings in the present study showed that the genetic-determined variables tend to converge in the final stages of maturation, following a linear evolution curve, although the between-players variability does not disappear. There were substantial improvements in performance during the basketball season, and these improvements continued to occur with older players. However, performance improvements tended to slow down, leveling-off when the players approach adult maturity status. Thus, variance can be explained by sport experience, notably less evident in tests where the movement of body mass over short distances is needed, such as the LD test.

This interpretation is of relevance applied to basketball player selection. The mean age at menarche was 11.82 (1.25) years, which is earlier than worldwide observations (Eveleth and Tanner, 1991), as well as on observations based on Brazilian data (Duarte, 1993). Hence, the present sample of female basketball players was, on average, advanced in maturity status expressed by mean age at menarche. This trend is consistent with observations in adolescent male basketball players where an overrepresentation of players with advanced maturity status has been noted (Carvalho et al., 2011b, 2013, 2018; Te Wierike et al., 2015; Torres-Unda et al., 2016). Hence, youth basketball coaches likely are not be considering the transient influence of maturation when interpreting young athletes' performance.

The interpretation of the random effects allows us to determine that all the evolution paths are linear even in tests that require explosive short-term strength. The importance of body mass and adiposity on functional performance are well known (Nevill et al., 2004), particularly in young populations (Barker and Armstrong, 2011). However, there was no substantial influence of body size on longitudinal changes in performance when aligning for chronological age or age at menarche in the present sample of female adolescent basketball players. Only adiposity had a negative influence on performance, which is consistent with longitudinal observations in non-athletic girls (Welsman and Armstrong, 2000; Armstrong et al., 2001). On the other hand, it should be expected that relative gains of body fat around 25 to 30% will occur by the end of puberty in the average adolescent girl (Matthews et al., 2006; Sherar et al., 2007; Mcmanus and Armstrong, 2011). Although young athletes tend to be leaner than non-athletic girl (Mcmanus and Armstrong, 2011), it appears that coaches should still need to consider pubertal body composition changes when interpreting female players performance development.

There was substantial variability between players across the 9-month competitive season exposure, however paths of performance development remained consistent across puberty. Differences between players at the beginning of the competitive seasons remained at the end of the season, although substantial variation on rates of changes across the season highlight the need for coaches to look at the players from an athlete-centered perspective. Furthermore, differences between players' rate of change in functional performance across competitive seasons during pubertal years appeared to be positively related to chronological age and biological age.

The alignment of chronological age with biological age and accumulated years of experience in the sport provide important information for youth sport organizers and coaches. Children and adolescents' growth with age follows a pattern that is genetically determined, albeit substantial between individuals' variation in both tempo and timing of growth between individuals (Malina et al., 2004). When the effects of maturation reach their end, about 15–16 years in average girls, all the players in the present sample, despite their variability, went through the same process. Hence, there is no need to artificially manipulate youth competitions in order to accelerate gains that sooner or later reach their peak and tend to flatten their improvement curve. At the same time, coaches need to be aware of the alignment of their interventions in preparation, providing the athlete with the training stimuli that match their readiness and knowing that there is no point in trying to force or accelerate those stimuli because the gains tend to slow down with the advance in chronological and biological age. These observations are of particular relevance given the recent calls promoting biobanding as a new paradigm for youth sports and training (Cumming et al., 2017; Rogol et al., 2018). The present results suggest the need to be cautious when interpreting the young athletes' performance, the need to consider athletes development over time, and to avoid decisions (competition groups, exclusion or promotion of athletes) based on snapshots using maturity status estimations that, at best, have limited validity (Malina et al., 2012; Malina and Koziel, 2014;Koziel and Malina, 2018).

We acknowledge that several limitations in the present study. The sample size is small and there was attrition between measurements. This may in part reflect the particular characteristics of context of the study (i.e., youth female basketball in Brazil), warranting caution when generalizing interpretations. Also, attrition is an important limitation in longitudinal studies during growth and training (Kemper, 2008). On the other hand, we only considered the follow-up of body dimensions and functional capacities in this study given the available time and context of assessment. Future studies may consider tracking also behavioral and in-game performance, but these pose considerable challenges when studying young players' development during pubertal growth. Nevertheless, the present data add valuable insights for the study of young female basketball players' physical and functional development. Moreover, Bayesian multilevel modeling was adopted to deal with the analytical challenges posed in a design with repeated observations within players over time, with different levels and sources of variation (within- and between-players). In contrast with traditional statistical approaches used in sports science, Bayesian multilevel modeling is a flexible and powerful approach to interpret young athletes' performance.

In summary, this study provides a description and interpretation about the development of functional performance across adolescence in female basketball players, accounting for the influence of growth, maturation and training on competitive basketball performance. It shows the need to account for chronological, biological and training experience, i.e., age in sport, and partition their influence on body size. Human growth follows a genetically determined pattern, despite substantial variation in both tempo and timing during puberty. When the effects of maturation reach their end, all the girls went through the same process. Hence, coaches, sport scientists, and others involved in the selection and development of youth basketball players should consider that there is no need to artificially manipulate youth competitions in order to accelerate gains that sooner or later reach their peak and tend to flat their improvement curve.

## Ethics Statement

This study was approved and carried out in accordance with the recommendations of the local Institutional Ethics Review Board. All participants were informed about the nature of the study, that participation was voluntary and that they could withdraw from the study at any time. Players and their parents/legal guardians provided written informed consent in accordance with the Declaration of Helsinki.

## Author Contributions

HC and CG were involved in the conceptualization of the study, data analysis, and the writing of the manuscript. RP and CF were involved in the conceptualization of the study and the writing of the manuscript. TL and AS were involved in the data assessment, and the writing of the manuscript. All authors contributed approved the final version of the manuscript.

### Conflict of Interest Statement

The authors declare that the research was conducted in the absence of any commercial or financial relationships that could be construed as a potential conflict of interest.
