# Supplementary material for: Longitudinal Changes of Functional Capacities Among Adolescent Female Basketball Players
Source: Front Physiol. 2019 Apr 4;10:339. doi: 10.3389/fphys.2019.00339 (PMC6459046; doi:10.3389/fphys.2019.00339)
Supplement: Supplementary file 1 [file Table_1.DOCX]

**Supplementary table 1**. Bayesian multilevel modeling for changes of functional performance between 12 and 17 years in female basketball players.

|  | Countermovement jump, cm | Line drill test, s | Yo-Yo IR1, m | Performance index, # |
| --- | --- | --- | --- | --- |
| *Population-level effects (90% Credible Interval)* | |  |  |  |
| Intercept | 26.18 (25.04 to 27.30) | 35.64 (35.17 to 36.12) | 537.0 (486.3 to 588.0) | 0.18 (-1.43 to 1.86) |
| Age centered | 1.42 (0.86 to 1.96) | -0.45 (-0.78 to -0.12) | 102.4 (71.7 to 132.7) | 3.65 (2.69 to 4.64) |
| Age centered^2^ | -0.24 (-0.48 to -0.00) | 0.16 (0.01 to 0.31) | - | - |
| *Group-level effects (90% Credible Interval)* | |  |  |  |
| *Level 1* *standard deviation (within player)* | |  |  |  |
| Within-individuals | 2.11 (1.88 to 2.35) | 1.25 (1.10 to 1.40) | 145.6 (126.8 to 158.3) | 3.75 (3.33 to 4.22) |
| *Level 2* *standard deviation (between players)* | |  |  |  |
| Intercept | 3.71 (2.96 to 4.58) | 1.43 (1.08 to 1.82) | 168.3 (130.7 to 214.7) | 5.33 (4.15 to 6.79) |
| Age centered | 0.68 (0.06 to 1.47) | 0.56 (0.06 to 1.09) | - | 1.13 (0.19 to 2.10) |

Note: age centered at 13.94 years

**Supplementary table 2**. Bayesian multilevel modeling for changes of functional performance by age of menarche in female basketball players.

|  | Countermovement jump, cm | Line drill test, s | Yo-Yo IR1, m | Performance index, # |
| --- | --- | --- | --- | --- |
| *Population-level effects (90% Credible Interval)* | |  |  |  |
| Intercept | 24.13 (22.35 to 25.81) | 36.44 (35.73 to 37.11) | 389.8 (306.5 to 466.5) | -6.34 (-9.28 to -3.54) |
| Distance to menarche | 0.80 (0.24 to 1.36) | -0.55 (-1.01 to -0.12) | 68.9 (41.7 to 96.5) | 2.84 (1.86 to 3.85) |
| Distance to menarche ^2^ | - | 0.08 (0.00 to 0.18) | - | - |
| *Group-level effects (90% Credible Interval)* | |  |  |  |
| *Level 1* *standard deviation (within player)* | |  |  |  |
| Within-individuals | 2.23 (2.00 to 2.49) | 1.30 (1.17 to 1.45) | 146.7 (130.9 to 164.8) | 3.75 (3.33 to 4.22) |
| *Level 2* *standard deviation (between players)* | |  |  |  |
| Intercept | 4.03 (3.11 to 5.12) | 1.56 (1.21 to 1.97) | 183.3 (140.2 to 233.5) | 6.59 (5.14 to 8.33) |
